# Lactic Acid Bacteria Enriched from Human Gastric Biopsies

**DOI:** 10.5402/2011/109183

**Published:** 2011-07-20

**Authors:** Elias Hakalehto, Terttu Vilpponen-Salmela, Kristiina Kinnunen, Atte von Wright

**Affiliations:** ^1^Finnoflag Oy, P.O. Box 262, 70101 Kuopio, Finland; ^2^Department of Biosciences, University of Eastern Finland, P.O. Box 1627, 70211 Kuopio, Finland; ^3^Harjula Hospital, P.O. Box 38, 70101 Kuopio, Finland

## Abstract

The purpose of this paper was to check if viable bacteria, in particular lactic acid bacteria (LAB), could be enriched from biopsies obtained from healthy gastroscopy patients. Gastric biopsies were obtained from 13 gastroscopy patients and subjected to an anaerobic or microaerophilic enrichment procedure utilizing the Portable Microbe Enrichment Unit (PMEU). Profuse microbial growth was observed in most cases. Samples plated on MRS showed high numbers of LAB. The most common species characterized were *Lactobacillus reuteri, Lact. salivarius*, and *Streptococcus salivarius*. The results demonstrate a continuous presence of viable LAB in healthy stomach. The species are similar to those traditionally used in food applications. The gastric LAB strains could have a potential in developing probiotic foods aimed specially on the upper part of the gastrointestinal tract.

## 1. Introduction

Although a diverse bacterial biota has been detected in the human esophagus [1], the human stomach has traditionally been considered as an almost sterile environment, where bacterial presence is limited and any bacterial growth is a result of pathological conditions such as *Helicobacter pylori *infection or as a result of acid-suppressive therapy [2–4]. 

While the bacterial densities in stomach undoubtedly are considerably lower than those detected in the ileum and colon, the possibility of acid-tolerant bacteria being continuously present on the gastric mucosal surfaces cannot be excluded. Lactic acid bacteria (LAB) are particularly interesting in this respect, because of the demonstrated positive health effects of certain LAB strains as probiotic microorganisms [5, 6]. The ability to survive and transiently colonize the gastrointestinal tract has been considered as one of the selection criteria for efficient probiotics [7], the acidic environment of the stomach being one of the hurdles a probiotic bacterium faces during the gastrointestinal passage. The duodenal conditions with relatively high concentrations of bile substances favor a typical flora almost devoid of LAB [8]. Therefore, LAB with a demonstrated ability to survive in the gastric conditions might be potential candidates for novel probiotic strains. 

## 2. Materials and Methods

### 2.1. Gastroscopies

Altogether, thirteen subjects were recruited for the study among patients coming to normal diagnostic gastroscopy. The subjects had a normal Western diet and did not receive any medication that might interfere with gastrointestinal microbiota. Fasting overnight was the standard routine of the procedure. Biopsies were taken from the cardial, antrum, and pyloric regions of the stomach. 

### 2.2. Enrichment

**Figure 1:**
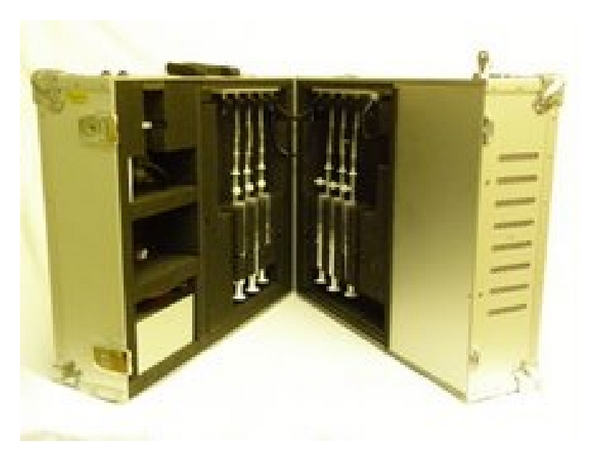
Portable Microbe Enrichment Unit (Samplion Oy).

The enrichment was done using the PMEU technology (Portable Microbe Enrichment Unit, Finnoflag Oy and Samplion Oy, Kuopio and Siilinjärvi, Finland, Figure 1) [9]. The biopsies were first aseptically immersed into test tubes containing 1.5 mL of thioglycollate medium (Difco, USA) and mixed carefully in an anaerobic glove box. Then the contents of the tubes were moved into 20 mL enrichment syringes. The syringes were containing 10 mL of fastidious anaerobe broth (Lab M, UK).

 In the present experiment the PMEU unit was adjusted to either anaerobic or microaerobic cultivation mode using continuous gas flow into the medium. The anaerobic gas mixture consisted of 80% N_2_, 10% CO_2_, and 10% H_2_ whereas the composition of the microaerobic gas flow was 5% O_2_, 10% CO_2_, and 85% N_2_. The pre-enrichment periods varied between 22 and 23.5 (for anaerobic cultures), and between 23.5 and 24 hours (for microaerobic cultures) at 37°C. 

The cultivable bacterial counts after pre-enrichment were studied by plating dilution series of the enrichment onto Petri dishes and cultivating them anaerobically at 37°C on Man-Rogosa Sharpe (MRS; Lab M, UK) agar (for lactic acid bacteria 72 h), on Plate Count (PC; Lab M, UK) agar (48 h), and on Wilkins Chalgren (Oxoid, UK) agar (for the *Bacteroides* group and other anaerobes, 72 h). Some samples were also used for plating onto MRS and PC agar without pre-enrichment and incubated both anaerobically and aerobically, the incubation times and temperatures being the same as in the platings from enrichment cultures. 

### 2.3. The Characterization of LAB

The cells from the most dominant isolates on the basis of colony morphology on MRS plates were Gram stained and observed microscopically. The Gram-positive rods or cocci were further subjected to tentative identification by API CH50 profiling (bioMérieux SA, France), followed by 16s DNA sequencing. Total genomic DNA was extracted from biopsy samples by using Charge Switch gDNA Mini Bacteria Kit (InVitrogen, USA). The primers used for the amplification were the archael 16S rDNA gene primers 27F (TCCGGTTGATCCTGCCGGAG) and 685R (TTACGGGATTTCACTCCTAC) yielding a 650 bp fragment. PCR amplifications were performed in a final volume of 50 *μ*L reactions using 1 *μ*L template DNA, 0,25 *μ*M of each primer, each dNTP at 0,2 mM, 1,5 mM MgCl_2_, 1x PCR buffer, and 1 U Go Taq Polymerase (Promega, USA). DNA was amplified for 40 cycles of 40 s at 94°C, 90 s at 56°C, and 40 s at 72°C followed by 10 min at 72°C. The amplified products were detected on 1% agarose gel and purified with NucleoSpin ExtractII kit (Macherey-Nagel, Germany). The PCR products were sequenced by Agova, Germany. The sequences were compared directly with the EMBL nucleotide database using the BLAST database sequence search engine. 

### 2.4. Ethical Aspects

The study plan was reviewed and approved by the Ethical Committee of the Kuopio University Hospital. 

## 3. Results

### 3.1. The Health Status of the Gastroscopy Patients

None of the patients recruited in the study proved to have *H. pylori* infection or any other obvious disturbance affecting the status of the gastric mucosa or the normal conditions of the stomach. 

### 3.2. The Bacterial Counts in the Enriched Samples

As can be seen in Table 1, high CFU counts of cultivable bacteria, typically around log 8 mL^−1^, were obtained during the enrichment. Without this step the CFU counts were typically near or below the detection limit of the plate counting (Table 2). However, even with enrichment, the bacterial numbers obtained from the same patient could vary considerably depending on the gastric location. For example, the biopsies from the antrum of patients 3 and 9 and the biopsy from the cardia of patient 3 showed no bacterial growth on some all or the media used, although the enrichment was applied. While in many cases the plate counts on Wilkins-Chalgren agar were several orders of magnitude lower than those on Plate count or MRS agar this was not universal. The counts on MRS tended to be rather close to those seen on Plate Count agar. 

### 3.3. The Species Distribution of the LAB Recovered from MRS Plates

The colonies on MRS plates typically represented morphologically one to three dominant types. These were further characterised and the species distribution, as indicated by the 16S rDNA sequence is given in Table 3. It can be seen that the majority of the recovered isolates were identified as *Streptococcus salivarius* or S*trep. sanguis* while the typical lactobacilli were *Lactobacillus reuteri *and* Lact. salivarius. *A single isolate of *Lact. casei *was also encountered. With the *Strep. salivarius *isolates the species indicated by the 16S rDNA sequence was actually the very closely related *Strep. thermophilus*, but since all the isolates were galactose-fermenting it was concluded that *Strep. thermophilus *is a more likely identification. 

## 4. Discussion

The PMEU technology has been previously successfully applied to enrich microorganisms from samples where their numbers are low [10–12] and the present study shows that the approach can be applied also to clinical specimen and that LAB, mainly streptococci and few lactobacilli, can be enriched from gastric biopsies. This species distribution differs from the typical intestinal LAB microbiota dominated by enterococci and a wide variety of lactobacilli [13]. Since the gastroscopy patients had fasted overnight, it is unlikely that the bacteria recovered represent transient food-associated microorganisms, but represent species of salivary origin. This is supported by the presence of *Strep. salivarius *and *Strep. sanguis, *which are normal oral bacteria and probably carried to stomach via ingestion. Also the possibility that the enrichment step selected certain species, thus creating bias in the species distribution, cannot be excluded.

On the basis of the limited data it cannot be stated, whether there is any preference for the bacterial presence regarding the different parts of the stomach (cardia, antrum, or pylorus).

Without the enrichment step the recovered bacterial counts were low, occasionally below the detection limit, indicating that cultivable mucosa-associated bacteria are rare, as can be expected. However, they can be found at least after the enrichment, and the question arises, whether they, including the ones originally ingested with food or saliva, represent some type of adaptation to gastric conditions. Since the focus of this study was on LAB, the colonies from Plate Count or Wilkins-Chalgren plates were not analysed further. These media were included to get an idea of the total amount of bacteria in the enrichment broth and whether there is some variability in the species composition that is reflected on differential growth on one of these media. The fact that the CFU counts on Wilkins-Chalgren medium were often (but not always) lower than on other media may indicate species differences between different individuals, particularly among anaerobes. 

Gastric environment is a challenge regarding the possibilities of probiotic therapy. For example, although some indication of beneficial effects of probiotics on *Helicobacter pylori* have been detected [14], the standard probiotic strains, being of faecal origin, probably are not optimal regarding the gastric or duodenal conditions. Strains derived from that region could have advantages in this respect. Additionally, gastric strains could also be useful because of their improved survival during the gastrointestinal passage, as already indicated in introduction. 

With a proper design of the enrichment conditions (composition and the pH of the medium) potential probiotic candidates could be isolated also from the gastric environment. 

## Figures and Tables

**Table 1:** The CFU counts recovered from the gastric biopsies.

Patient number	Enrichment conditions	log CFU mL^−1^ medium^a^								
		Site of the biopsy								
		Cardia			Antrum			Pylorus		
		Plate count agar	MRS agar	Wilkins Chalgren agar	Plate count agar	MRS agar	Wilkins Chalgren agar	Plate count agar	MRS agar	Wilkins Chalgren agar
1	Anaerobic	8.9	9.0	8.0	8.5	8.7	8.1	8.5	8.7	8.0
2	Anaerobic	8.3	8.1	7.0	<5.0	5.3	<5.0	<5.0	<5.0	<5.0
3	Anaerobic	8.2	8.3	<5.0	<1.0	<1.0	<1.0	<1.0	<1.0	<1.0
4	Anaerobic	9.2	8.3	5.9	8.9	7.5	<5.0	<1.0	<1.0	<1.0
5	Microaerophilic	8.0	8.4	7.6	8.2	8.3	6.6	8.2	8.2	6.6
6	Microaerophilic	8.0	8.0	8.0	8.4	8.1	7.1	8.5	8.5	8.1
7	Microaerophilic	8.5	8.0	8.5	8.4	8.3	8.3	7.8	8.5	8.4
8	Microaerophilic	8.6	8.8	4.7	8.5	8.6	5.3	8.5	8.5	6.9
9	Microaerophilic	8.1	8.1	8.1	<1.0	5.8	<1.0	8.3	8.1	5.8
10	Microaerophilic	8.6	8.1	8.0	7.8	8.0	5.7	8.4	8.4	6.6
11	Microaerophilic	6.1	6.0	<5.0	8.1	8.5	6.4	8.0	7.6	<5.0
12^b^	Microaerophilic	9.3	8.6	nd^c^	8.3	8.3	nd	7.9	8.0	nd
13^b^	Microaerophilic	<2.0	6.2	nd	6.6	6.6	nt	6.3	6.4	nd

^a^< in front of the log means values below the detection limit in the particular experiment.

^b^The plating and incubation of these samples were done in aerobic conditions.

^c^nd = not done.

**Table 2:** Background values of CFU counts from the enrichment culture before the enrichment step.

Patient number	Plating conditions	log CFU mL^−1^ medium^a^					
		Site of the biopsy					
		Cardia		Antrum		Pylorus	
		Plate count agar	MRS agar	Plate count agar	MRS agar	Plate count agar	MRS agar
11	Anaerobic	6.1	6.0	5.3	4.2	2.3	3.1
12	Anaerobic	3.0	<2.0	2.8	2.8	3.1	3.3
	Aerobic	2.2	<2.0	3.0	3.2	3.5	3.0
13	Anaerobic	<2.0	<2.0	<2.0	<2.0	<2.0	<2.0
	Aerobic	<2.0	<2.0	3.1	<2.0	<2.0	<2.0

^a^< in front of the log means values below the detection limit in the particular experiment.

**Table 3:** The identified LAB species enriched from the biopsies obtained from different gastric sites.

Patient number	Cardia	Antrum	Pylorus
1	*Ent. faecalis *(2)^a^	*Lact. salivarius (2) *	*Lact. salivarius *
	*Step. salivarius *		
	*Lact. salivarius ssp. salivarius*		
2	*Strep. salivarius *(2)		

4	*Strep. sanguinis *(2)	*Strep. salivarius*
	*Strep. salivarius*	
	*Ent. faecalis *	

5	*Strep. salivarius*	*Strep. salivarius*	*Lact. salivarius *

6		*Lact. reuteri *	*Lact. reuteri *
		*Lact. casei*	

7		*Lact. reuteri *(3)	*Lact. reuteri *
		*Strep. salivarius*	

8		*Step. sanguinis *	*Lact. reuteri *(2)

9	*L. lactis ssp. lactis (2) *		* Strep. salivarius *

10	*Strep. sanguinis *	*Strep. sanguinis *	*Strep. sanguinis *

11	*Strep. salivarius* (2)	*Strep. salivarius* (2)	*Lact. reuter*i (3)

12	*Strep. salivarius*	*Strep. salivarius *(3)	*Strep. salivarius*
		*Lact. reuteri *(2)	

13	*Strep. salivarius *(2)		*Strep. salivarius*

^a^The number in parenthesis is the number of isolates if more than one.
